# Pegylated liposomal doxorubicin in patients with metastatic triple-negative breast cancer: 8-year experience of a single center

**DOI:** 10.1186/s43046-020-00034-4

**Published:** 2020-04-27

**Authors:** Salah Mabrouk Khallaf, Jasmine Roshdy, Abeer Ibrahim

**Affiliations:** Medical Oncology Lecturer, South Egypt Cancer Institute, Assiut University, Assiut, 71511 Egypt

**Keywords:** Metastatic breast cancer, Triple-negative, TNBC, Pegylated liposomal doxorubicin, PLD, Chemosensitivity, Chemosensitive tumor

## Abstract

**Background:**

The known efficacy of doxorubicin in metastatic breast cancer is countered by its dose-limiting myelosuppression and cardiotoxicity. Pegylated liposomal doxorubicin (PLD) was discovered to overcome these problems. But the data regarding its use in metastatic TNBC (triple-negative breast cancer) is still insufficient. Our study aimed to assess the factors affecting the outcome of the patients with metastatic TNBC who received PLD.

**Results:**

During a period of 8 years (January 2011–December 2018), we analyzed 39 eligible patients. The disease control rate (DCR) was 51.3%. Among all the analyzed factors, two of them significantly affected DCR. The first factor was the chemosensitivity to prior anthracycline. As patients with chemosensitive disease had higher DCR than those with the chemoresistant disease (*P* = .001). The second factor was the number of prior lines of chemotherapy. As the patients who received two prior lines had a higher DCR than those who received three lines or more (*P* = .023). Chemosensitivity was the only significant independent factor for DCR (odds ratio = .095, *P* = .008).

For the studied patients, the median progression-free survival (PFS) was 7 months. The anthracycline-chemosensitivity was the only significant independent prognostic factor for PFS (*P* = .002). The median overall survival (OS) was 12 months. There was a marginally significant effect of anthracycline-chemosensitivity on OS (*P* = .052).

**Conclusion:**

The anthracycline-chemosensitivity is an independent predictive and prognostic factor for the patients with metastatic TNBC receiving PLD. In developing countries, PLD should be reserved for the patients whose tumors are anthracycline-chemosensitive.

## Background

Breast cancer is the most common malignancy diagnosed in women worldwide [1]. Triple-negative breast cancer (TNBC) accounts for about 15% of all breast cancer [1]. About one third of patients with TNBC have a metastatic disease. It is either de novo or recurrent metastasis [2]. A triple-negative phenotype is defined by the lack of protein expression of the estrogen and progesterone receptors and the absence of HER2 protein overexpression [3].

When indicated, the immunotherapy and polyadenosine diphosphate-ribose polymerase inhibitors (PARPis) are the most active agents in the treatment of metastatic triple-negative breast cancer (TNBC) [4]. The immunotherapy with atezolizumab is indicated in combination with nab-paclitaxel in the treatment of patients with advanced TNBC. It is only useful in patients whose tumors express programmed cell death ligand 1 (PD-L1 ≥ 1% on the tumor-infiltrating immune cells) [5]. Two PARPis, olaparib and talazoparib, are approved for the treatment of metastatic HER2-negative patients with germline BRCA1- or BRCA 2-mutation. This is based on two independent phase III randomized controlled trials [6]. In developing countries, the high cost of the immunotherapy and PARPis limited their use. Hence, chemotherapy is still a treatment option for those patients. There are several active agents with established single-agent activity. Doxorubicin is among these active agents [7]. Battisti et al. (2018) conducted a recent real-world review about the role of systemic therapy for the advanced TNBC in 186 eligible patients. They investigated seven categories of systemic therapy including anthracyclines and their combinations. They found that ECOG (Eastern Cooperative Oncology Group) performance status and disease-free interval were the only independent factors for disease-free survival and overall survival [8]. The known efficacy of doxorubicin in metastatic breast cancer (MBC) is countered by its dose-limiting myelosuppression and cardiotoxicity [9, 10]. This cardiotoxicity is dose-related. Hence, the recommended cumulative dose of doxorubicin should not exceed 450–500 mg/m^2^ [11–13]. To minimize doxorubicin toxicity while preserving its efficacy, a new formulation of this drug was invented by its capsulation with polyethylene glycol-coated liposome. This formulation is called pegylated liposomal doxorubicin (PLD). Many studies documented the efficacy of PLD in patients with MBC [7, 9, 11, 13–18]. Out of the prior studies, two scientific papers (one abstract [17] and one case report [18]) reported the role of PLD in metastatic TNBC, but without addressing the predictor factors for the outcome. Our study aimed to assess the effect of patients’ demographics and tumor characteristics on the outcome of patients who received PLD for metastatic TNBC.

## Methods

### Study design

**Fig. 1:**
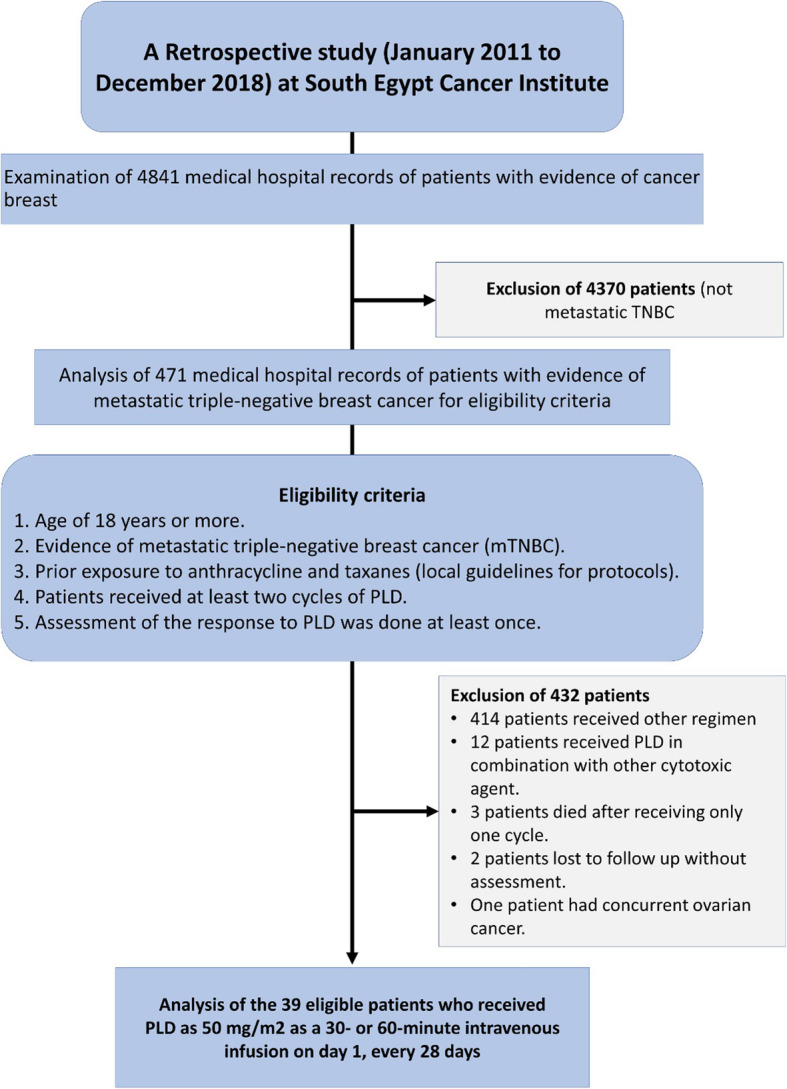
CONSORT diagram

During a period of 8 years (January 2011 to December 2018), we analyzed the medical hospital records of patients with evidence of metastatic breast cancer (MBC). The eligibility criteria included all the followings: age of 18 years or more; evidence of metastatic TNBC; prior exposure to anthracycline and taxanes either in adjuvant, neoadjuvant, or palliative setting (as the local guidelines for protocols permitted PLD as a 3rd or 4th palliative line of chemotherapy during the time of this study); and patients receiving at least two cycles of PLD; and assessment of the response to PLD that was done at least once. The exclusion criteria included any of the following items: double malignancy other than bilateral breast cancer, patients received PLD in combination with another cytotoxic agent, or patients lost to follow-up without any assessment. The details are shown in Fig. 1 (CONSORT diagram).

### Patient stratification

We stratified the analyzed patients according to the patient-related factors: the age group (less than 50 years vs. 50 years or more), the Eastern Cooperative Oncology Group (ECOG) performance status (grade 0/1 vs. grade 2), and the menopausal status (pre/perimenopausal vs. postmenopausal); the tumor-related factors: the pathological type (ductal vs. lobular vs. other types), the pathological grade (I/II vs. III) based on the Nottingham grading system [19], the type of metastatic disease (de novo vs. recurrent), and the number of metastasized organ/s (solitary vs. multiple); and the therapy-related factors: the number of prior lines (two vs. three or more) and the chemosensitivity to prior anthracycline (chemosensitive vs. chemoresistant) (Table 1).

**Table 1:** *P*atients’ demographics and tumor characteristics (*N* = 39)

Characteristic	*N*^o^	%
Age (years)		
˂ 50 years	29	74.4
≥ 50 years	10	25.6
Mean	45.9	
Standard deviation (SD)	6.9	
ECOG PS		
0, I	11	18.3
II	28	81.7
Menopausal status		
Pre/perimenopausal	26	66.6
postmenopausal	13	33.3
Pathological type		
IDC	34	87.2
ILC	4	10.3
Others	1	2.6
Tumor grade		
Grade I or II	33	84.6
Grade III	6	15.4
Type of metastasis		
De novo MBC	8	20.5
Recurrent MBC	31	79.5
No of metastasized organ/s		
One	5	12.8
Two or more	34	87.2
Order of PLD line		
3rd palliative	13	33.3
4th palliative or more	26	66.6
Chemosensitivity		
Chemosensitive	14	35.9
Chemoresistant	25	64.1
Chemotherapy cycles		
Total number	222	
Mode	3	
Range	3–18	

Abbreviations: *ECOG* Eastern Cooperative Oncology Group, *IDC* invasive ductal carcinoma, *ILC* infiltrating lobular carcinoma, *Her 2* human epidermal growth factor receptor 2, *PS* performance status, *SD* standard deviation

### Definition of anthracycline-resistance and anthracycline-sensitivity

Anthracycline-resistance was defined when progression or recurrence occurs during the anthracycline-based treatment, relapse within 12 months after the last adjuvant or neoadjuvant anthracycline cycle, or relapse within 6 months after the last palliative anthracycline cycle in patients achieving complete remission, whereas the anthracycline-sensitive tumor (also called anthracycline non-resistant) is defined when the prior criteria of the chemoresistant tumor are not fulfilled. Tumor with either complete response, partial response, or stationary response was considered chemosensitive as long as it did not progress or recur [16, 20–22].

### Treatment and tool of assessment

PLD was given according to the local protocol: 50 mg/m^2^ as a 60-min intravenous infusion on day 1, repeated every 28 days. Treatment was continued until maximal response, unacceptable toxicity, or patient’s refusal to continue. Dose modifications were done according to the local protocol. The response rate was based on the Response Evaluation Criteria in Solid Tumors (RECIST 1.1) [23].

### Statistical analysis

The primary endpoints were the disease control rate (DCR) and progression-free survival (PFS). DCR was defined as a sum of rates of stable disease [SD] for more than 6 months, complete response (CR), and partial response (PR). PFS was defined as the time from the start of treatment to disease progression or death from any cause or date of the last follow-up, whichever came first. The secondary endpoints were overall survival (OS) and safety profile. Overall survival was defined as the time from the start of treatment to date of death from any cause, or date of the last follow-up, whichever came first). Safety was assessed according to the guidelines of the Common Terminology Criteria for Adverse Events V. 4.03 (CTCAE) [24]. Univariate analysis was used through the presentation of continuous variables as main and standard deviation (SD). Categorical variables (nominal and ordinal) are presented as frequency and percentage. Bivariate analysis was done to compare categorical variables using the chi-square test or Fisher’s exact test when appropriate. Multivariate analysis by using logistic regression analysis was used to determine the independence of the significant factors for DCR. Kaplan-Meier method was used to estimate the survival time distribution and the median survival of studied patients. Multivariate analysis by using Cox regression analysis was used to determine the independence of the significant factors for survival. A *P* value less than 0.05 was considered as a cutoff of significance. SPSS version 21.0 (SPSS Inc. Chicago, IL, USA) was used in the storage and analysis of data [25].

## Results

### Patients’ demographics and tumor characteristics

As illustrated in the CONSORT (Fig. 1), we examined 4841 medical records of the patients with breast cancer; 471 out of them had MBC. Only 39 patients were eligible for the study. The details of patients’ demographics and tumor characteristics are shown in Table 1. Most patients were pre/perimenopausal (66.6%) and had an ECOG performance status of grade 2 (81.7%). The most common pathological type was IDC (87.2%). Most metastases occurred as a recurrent disease (79.5%). We found that fourteen patients (35.9%) had an anthracycline-chemosensitive tumor, while the remaining 25 patients had a chemoresistant tumor. One third of the cases received PLD as a 3rd line palliative chemotherapy, while the remaining two third received it as a 4th line or more.

### Response rates

Based on RECIST V. 1.1, we found that twenty patients (51.3%) had DCR (sum of the rates of the complete remission in three cases, the partial remission in five cases, and the stable disease in twelve cases) (Table 2).

**Table 2:** Disease control rate and overall response rate according to RECIST Criteria V 1. 1

Characteristic	ORR			DCR		
	No/total	%	*P* value*	No/total	%	*P* value*
Chemosensitivity			.002			.001
Chemosensitive tumors	6/14	42.9		12/14	85.7	
Chemoresistant tumors	1/25	4.0		8/25	32.0	
Number of prior lines						.023
Two lines	5/13	38.5	.018	10/13	76.9	
More than two lines	2/26	7.7		10/26	38.5	
Age (years)					51.7	.925
˂ 50 years	5/29	17.2	.845	15/29	50.0	
≥ 50 years	2/10	20.0		5/10		
Menopausal status				6/13	46.2	.651
Pre/perimenopausal	5/26	19.2	.768	14/26	53.8	
Postmenopausal	2/13	15.4				
ECOG PS				5/11	54.5	.798
0, I	1/11	9.1	.649	14/28	50.0	
II	6/28	21.4				
Pathological type				16/34	52.9	.351
IDC	5/34	14.7	.084	1/4	25.0	
ILC	1/4	25.0		1/1	100.0	
Others	1/1	100.0				
Tumor grade				17/33	51.5	.946
Grade I or II	6/33	18.2	.929	3/6	50.0	
Grade III	1/6	16.7				
Type of metastasis				6/8	75.0	.132
Denovo MBC	2/8	25.0	.617	14/31	45.2	
Recurrent MBC	5/31	16.1				
No of metastasized organ/s				2/5	40.0	.676
One	1/5	20.0	.898	17/34	50.0	
Two or more	6/34	17.6				

*P value calculated by Pearson chi-square test or Fisher’s exact test when appropriate though crosstabulation

Abbreviations: *CR* complete response, *DCR* disease control rate, *ORR* overall response rate, *PR* partial response, *RECIST* response evaluation criteria in solid tumors, *SD* stable disease

With univariate analysis, we found that two out of the analyzed factors had significantly affected DCR. The first one was the anthracycline chemosensitivity. Interestingly, the patients with chemosensitive tumors had a higher DCR when compared to those with chemoresistant ones (85.7% vs. 32%, respectively, *P* = .001). The second factor was the number of prior chemotherapy lines: patients who received only two prior lines had better DCR than those who received more than two prior lines (76.9% vs. 38.5%, respectively; *P* = .023).

With multivariate analysis using logistic regression, the chemosensitivity was an independent significant predictor factor for DCR; the odds ratio was .095 (95% CI .015–.533) for patients with chemosensitive tumors when compared to those with chemoresistant tumors (*P* = .008).

### Survival outcome

#### Progression-free survival (PFS)

**Fig. 2:**
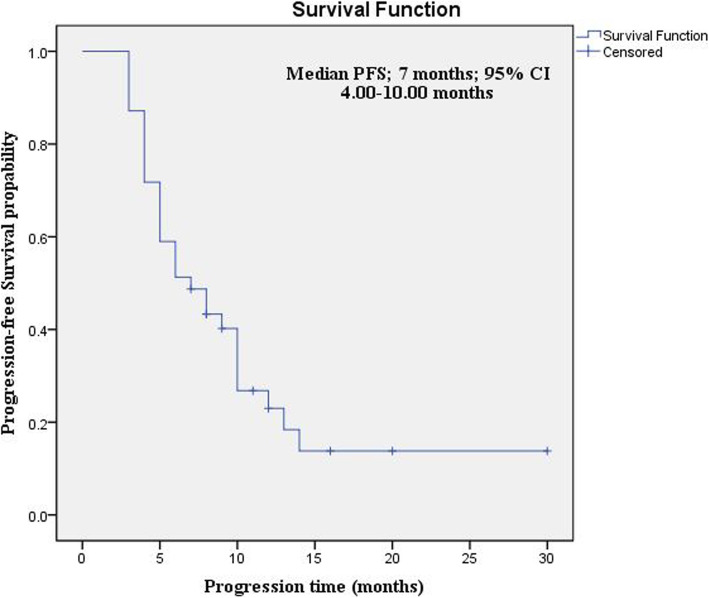
Kaplan-Meier plot: progression-free survival. CI confidence interval, PFS progression-free survival

**Fig. 3:**
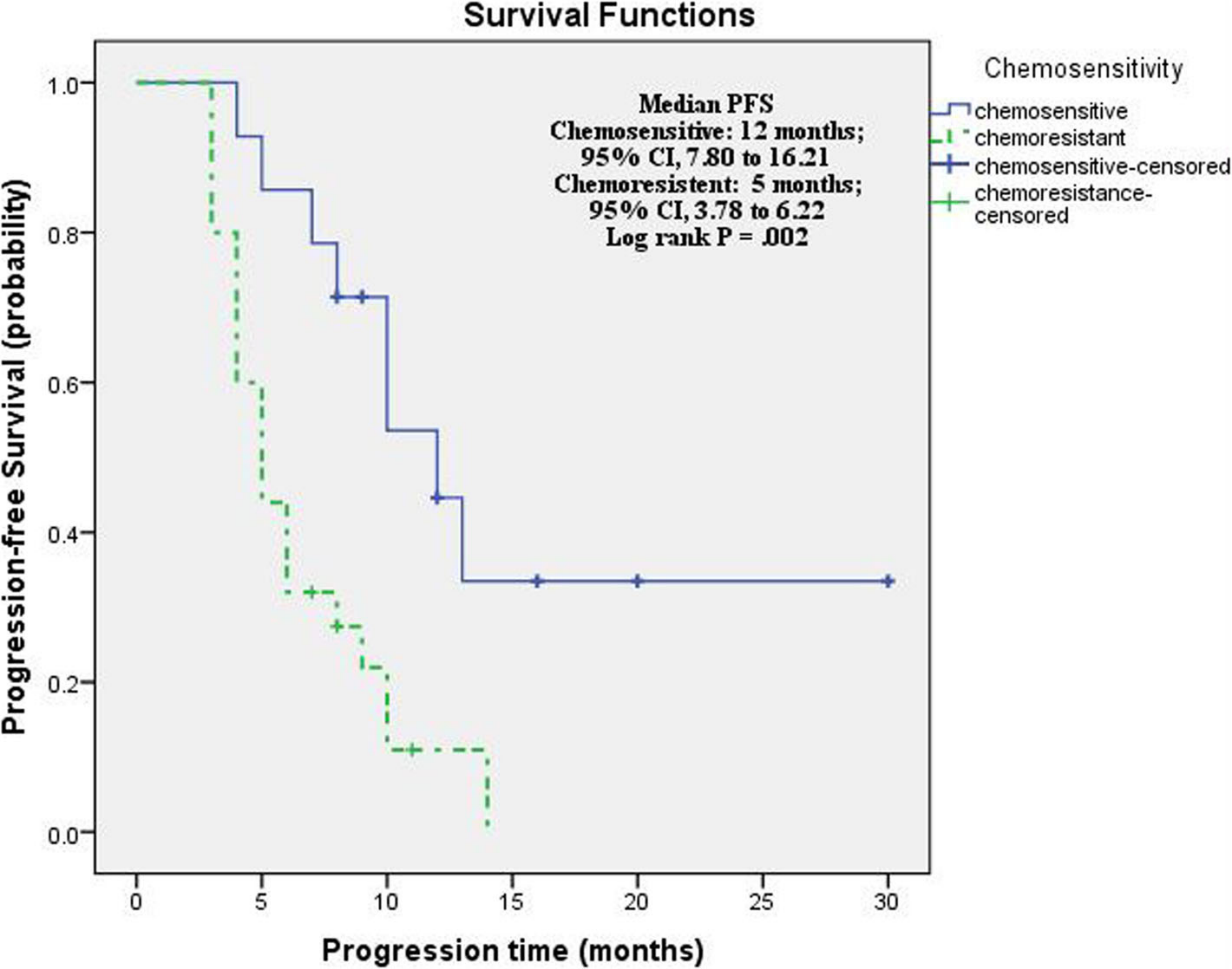
Kaplan-Meier plot: progression-free survival according to chemosensitivity to prior anthracycline. CI confidence interval, PFS progression-free survival

The median PFS was 7 months, with 95% CI 4.00–10.00 months (Fig. 2). The anthracycline chemosensitivity and type of metastatic disease were the factors that significantly affected the PFS. The median PFS was more than twice as long for patients with a chemosensitive tumor when compared to those with chemoresistant tumor (median PFS was 12 months, 95% CI 7.80–16.21 versus 5 months; 95% CI 3.78–6.22, respectively; *P* = 002) (Fig. 3).

The significance of the effect of the type of metastatic disease on median PFS is marginal (*P* = .053). As patients with de novo metastasis had a median PFS of 10 months; 95% CI 7.85–12.15 months, compared to 6 months, and 95% CI 4.65–7.35 months for those with recurrent metastatic disease.

Multivariate analysis was done through Cox regression analysis. We found that chemosensitivity was the only independent factor for longer PFS (hazard ratio .34; 95% CI .15–.80; *P* = .013).

#### Overall survival (OS)

**Fig. 4:**
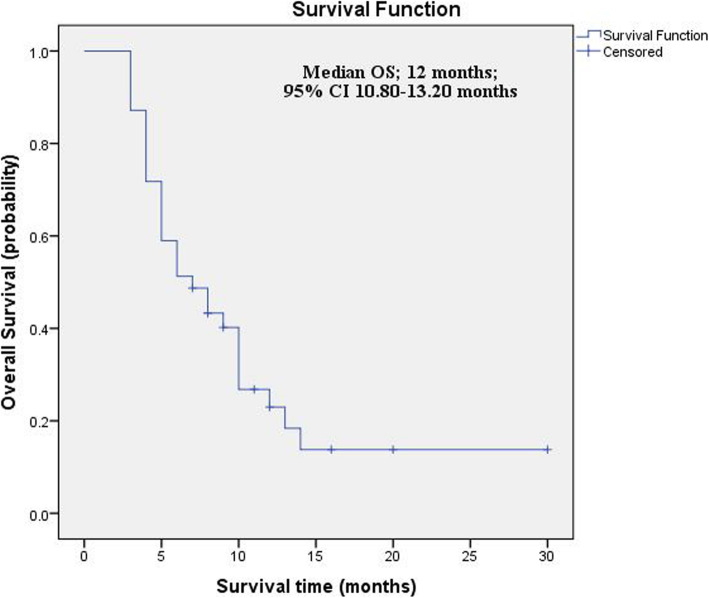
Kaplan-Meier plot: overall survival. CI confidence interval, OS overall survival

**Fig. 5:**
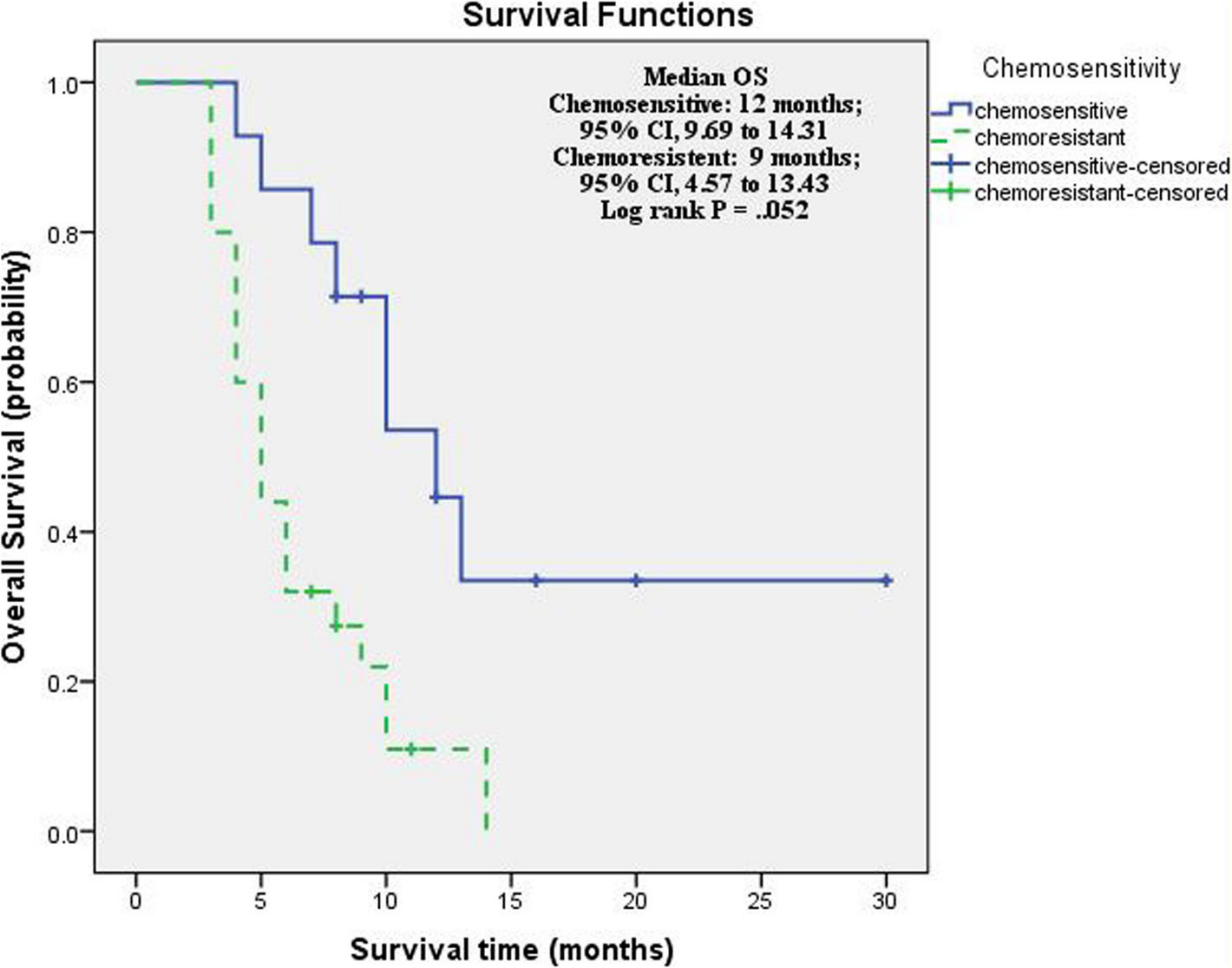
Kaplan-Meier plot: overall survival according to chemosensitivity to prior anthracycline. CI confidence interval, OS progression-free survival

The median OS was 12 months; 95% CI 10.80–13.20 months (Fig. 4). Our data demonstrated a marginally significant effect of chemosensitivity on the median OS. Patients with the chemosensitive disease had slightly better median OS (12 months; 95% CI 9.69–14.31) when compared to that of patients with the chemoresistant disease (9 months, 95% CI 4.57–13.43; log-rank *P* = .052) (Fig. 5).

#### Safety

All cases received 222 cycles of PLD with a range of 3 to 18 cycles. Dose modifications were done in five cases (12.8%), while we could not continue that treatment in two cases (5.1%) due to palmar-plantar erythrodysesthesia (PPE) and hypersensitivity (one case for each adverse effect). Table 3 shows the main adverse effects, the majority were grades 1 or 2; with higher rates of non-hematological than hematological toxicities. For all grades, the most common non-hematological toxicity was PPE (33.3%), then stomatitis (20.5%), followed by mucositis and diarrhea (15.4% for each). For severe toxicity (grade 3 or more), PPE (10.3%) was the most common non-hematological toxicity, followed by mucositis and diarrhea (5.1% for each). No grade 3/4 cardiac toxicity was reported. Grade 3/4 hematological toxicity was relatively uncommon; 7.7% for leukopenia and 5.1% for each anemia and neutropenia. Only one case (2.6%) developed febrile neutropenia. There was no treatment-related death.

**Table 3:** Toxicity of pegylated liposomal doxorubicin in the studied patients

Event	All grades	Grade III/IV
	No (%)	No (%)
**Non-hematological**		
PPE	13 (33.3)	4 (10.3)
Stomatitis	8 (20.5)	2 (5.1)
Mucositis	6 (15.4)	1 (2.6)
Diarrhea	6 (15.4)	2 (5.1)
Constipation	2 (5.1)	0 (0)
Anorexia	4 (10.3)	0 (0)
Nausea	4 (10.3)	0 (0)
Vomiting	5 (12.8)	0 (0)
Hypersensitivity	5 (12.8)	1 (2.6)
Fatigue	4 (10.3)	1 (2.6)
Alopecia	3 (7.7)	0 (0)
Cardiac toxicity	2 (5.1)	0 (0)
**Hematological**		
Anemia	6 (15.4)	2 (5.1)
Leukopenia	4 (10.3)	3 (7.7)
Neutropenia	4 (10.3)	2 (5.1)
Thrombocytopenia	2 (5.1)	0 (0)

Abbreviations: *PPE* palmar plantar erythrodysesthesia

## Discussion

To the best of our knowledge, this study is the first one that specifically investigated the factors affecting the outcome of patients with metastatic triple-negative breast cancer (TNBC) receiving PLD. In this retrospective study, we analyzed the data of 39 patients with metastatic TNBC treated with PLD. In our results, DCR was 51.3%. Univariate analysis revealed that two out of the examined factors significantly affected DCR. These factors are chemosensitivity (chemosensitive tumor is better; 85.7% vs. 32%, respectively; *P* = .001) and the number of prior lines (two lines are better; 76.9% vs. 38.5%, respectively; *P* = .023). With multivariate analysis, chemosensitivity was the only independent predictor factor for DCR with an odds ratio of .095, 95% CI .015–.533, and *P* = .008.

The efficacy of PLD in metastatic TNBC is reported only in a Spanish study constructed by Martin-Romano et al. (2018), who enrolled only 15 patients with TNBC out of a total of 122 patients with MBC receiving PLD with gemcitabine [17]. They do not report the response rate for TNBC, but they report that ORR of 31.1% and DCR of 63.0% for all 122 patients. Although Martin-Romano et al. reported a higher response rate when compared to our result. This comparison is weak because they did not report the predictor factors of that response in TNBC. There is a case report paper published by Franchina et al. (2012), who reported the activity of pegylated liposomal doxorubicin in combination with gemcitabine in triple-negative breast cancer with skin involvement [18]. This result may raise the future thinking of the study of pegylated liposomal doxorubicin in combination with gemcitabine in TNBC. Still, it does not serve our idea about the predictive factors for the response to PLD in patients with TNBC.

The median PFS for our patients is 7 months, with a 95% CI of 4.00–10.00 months. We found that the chemosensitivity is the only independent significant prognostic factor affected the median PFS; patients with chemosensitive tumor had more than double median PFS of those with chemoresistant tumor (12 months, 95% CI 7.80–16.21 for patients with chemosensitive tumor vs. 5 months, 95% CI 3.78–6.22 for those with a chemoresistant tumor, *P* = .002) with hazards ratio of .34, 95% CI .15–.80, and *P* = .013 by Cox regression analysis. As regards the effect of chemosensitivity on median PFS, there are reports on MBC in general, not specifically TNBC. Consistently with our findings, Al-Batran et al. [21] who reported a higher PFS in patients having a non-anthracycline-resistant disease than that in patients having an anthracycline-resistant disease (2.8, 95% CI 1.9–7.1 for the patient with anthracycline-resistant disease vs. 3.7, 95% CI 2.8–7.8 for the patient with the non-anthracycline-resistant disease; however, *P* value was not reported). Also, Keller et al. [22] indirectly concluded that the anthracycline resistance lowered median PFS; they stated that PLD is superior to vinorelbine in patients with non-anthracycline-resistant MBC (median PFS, 3.7 vs. 2.6 months, respectively; HR, 1.26; 95% CI 0.87–1.82) and comparable to vinorelbine in patients with anthracycline-resistant MBC (median PFS, 2.6 vs. 2.6 months, respectively; HR, 1.14; 95% CI 0.70–1.83). We used the term anthracycline-chemosensitive instead of non-anthracycline-resistant, which are quite similar. From the previous results, the anthracycline-chemosensitivity is the most important prognostic factor for patients with TNBC receiving PLD.

For our patients, the median OS is 12 months and 95% CI 10.80–13.20 months. There is a numerical, but not a statistically significant effect of chemosensitivity on the median OS in favor of chemosensitive disease (12 months, 95% CI 9.69–14.31) compared to chemoresistant disease (9 months, 95% CI 4.57–13.43; log-rank *P* = .052). Fiegl et al. [26] studied the prognostic factors in patients who received PLD for MBC. These factors were the number of metastatic sites, the number of prior chemotherapies, the prior endocrine therapy, and the prior anthracycline [26]. In contrast to our result, they reported that the occurrence of a greater number (≥ 4) of metastatic sites is the only independent risk factor for shorter OS (hazard ratio 2.78, 95% CI 1.75–4.42; *P* < .001) [26]. This contrast may be due to different patients’ subgroups regarding the number of metastatic sites (one site vs. two or more sites for our patients while less than four sites vs. four or more metastatic sites for their study). Also, this may be due to different biological subgroups, as mentioned by Battisti et al. (2018) who published a recent real-world review about systemic therapy for advanced TNBC. They reported that TNBC is classified into six molecular subtypes [8]. Battisti et al. reported results that may emphasize our findings. They found that patients with disease-free interval (DFI) more than 12 months (these patients are similar to a chemosensitive group in our study) are associated with better progression-free survival and overall survival when compared to those with DFI < 12 months [8]. The data about the prognostic factors for OS in patients with TNBC receiving PLD is still insufficient. This lack of data may be due to inadequate data about this group of patients in previous studies and a relatively small sample size of our research.

From our study and the prior studies, the severe cardiac toxicity (grade 3/4) of PLD is rare (0.0% in our study, the study of Al-Batran et al. [21], and the study of Keller et al. [22]; 1% in Harbeck et al. study [16]; and < 1% in Huober et al. study [15]). This rare incidence of severe cardiac toxicity enables the clinicians to administer higher cumulative doses of PLD than that of conventional doxorubicin. There is no reliable data about the cumulative dose of PLD, and most of the previous studies continued the treatment with it till progression or unacceptable toxicity. Al-Batran et al. [21] administered up to 12 cycles without recording any event of severe cardiac toxicity like our study in which the maximal number of the cycles was 18 cycles. Even more, Harbeck et al. [16] reached up to 24 cycles of PLD with only a 1% incidence of grade 3/4 cardiac toxicity. The dose-limiting of the PLD is palmar-plantar erythrodysesthesia (PPE). In our study, it was the most common adverse effect (33.3% for all grades and 10.3% for grade 3/4 toxicities). The occurrence of grade 3/4 PPE varies between the studies according to the reported data (39% by Harbeck et al. [16], 19% for Keller et al. [22], 6.4% by Al-Batran et al. [21], 6% by Huober et al. [15], and 1% by Fiegl et al. [26]). This variance may be due to different patients’ characteristics and PLD dosage and schedule. Hematological toxicity was less common than non-hematological toxicity. Grade 3/4 neutropenia (5%) was the most common, followed by anemia and leukopenia (4% for each). There is no treatment-related death.

## Conclusion

The anthracycline-chemosensitivity is an independent predictive and prognostic factor for patients with metastatic TNBC receiving PLD. In developing countries, PLD should be reserved for the patients whose tumors are anthracycline-chemosensitive.

## Supplementary information

**Additional file 1.**

## Data Availability

All data generated or analyzed during this study are included in this published article [and its supplementary information files].

## Abbreviations

CIs: Confidence intervals
CR: Complete response
CTCAE: Common Terminology Criteria for Adverse Events
DCR: Disease control rate
ECOG: Eastern Cooperative Oncology Group
Her2: Human epidermal growth factor receptor 2
HRs: Hazard ratios
IDC: Invasive ductal carcinoma
ILC: Infiltrating lobular carcinoma
MBC: Metastatic breast cancer
OS: Overall survival
PARPis: Polyadenosine diphosphate-ribose polymerase inhibitors
PFS: Progression-free survival
PLD: Pegylated liposomal doxorubicin
PPE: Palmar-plantar erythrodysesthesia
PR: Partial response
SD: Stable disease
TNBC: Triple-negative breast cancer
vs.: Versus
